# Medical and Physical Intelligence

**Published:** 1799-12

**Authors:** 


### MEDICAL AND PHYSICAL
### *INTELLIGENCE,*
### ( Original and Selected. )

---

### *Observations on Soda, the Alkaline Basis of Animal Gall and of Sea-Salt, &c. by Dr.* MITCHILL *of New-York.*

[ Concluded from our last Number, p. p. 383—385. ]

SODA alone then can preserve the flesh of an animal from corruption; and if mere preservation was all that was intended, this alkali would, by itself, answer the purpose; and so would pot-ash. But there is some quality in the muriatic acid which makes the compound, which it forms with soda, a far preferable substance to prevent the corruption of meat intended to be eaten.

In order to understand what effect the muriate of soda has, it will be proper to consider what change the piece of meat in the larder was undergoing, which could be arrested by the sea-salt. The flesh of animals (I mean, particularly, the muscular or lean parts) may be considered as verging towards a putrefactive state from the time the fibres lose their irritability, and become rigid. And one of the signs of incipient putrefaction, under circumstances favourable to that process, is sometimes a *sourness* or *production of acidity* in the substance; (4 Fourcroy, chap. xvi.) and this acidity is inherent in the meat, and quite another thing from fixed air. What I mean is the *septic acid, which somtimes poisons dissectors, and which, when volatilized into gas, and diffused through the atmosphere, causes violent endemic distempers.* When produced, it is formed early in the putrefactive process, and before the whole flesh has undergone total disorganziation. But, fortunately for mankind, it is not *always* engendered during the putrefaction, even of those substances which contain its basis, septon. This radical frequently escapes in the form of azotic air, without combining with oxygen at all, and, in such cases, the exact matter of pestilential fluids is not formed.

In cases where the septic acid is formed in salted meat, the muriat of soda is decompounded; and while the alkali attracts the destructive septic acid to itself, it parts with the muriatic. The flesh, in proportion as it becomes impregnated with this new and preserving ingredient, progresses, afterwards, slowly towards decomposition, because it is enveloped with a liquid acting feebly upon it, and upon which it can exert, in its turn, but a feeble action. Hence, when beef, or any other lean meat, is plentifully charged and surrounded with salt, there is enough of soda to neutralize the acid of putrefaction, should any be formed, and there is also a corresponding proportion of the spirit of salt disengaged; from which latter proceeds, in a certain degree, the agreeable smell and pleasant taste of well-salted provisions.

To

To preſerve the fleſh of animals, there muſt, therefore, be not only ſalt enough in the barrel, but this ſalt muſt be applied to every part of the meat, by cutting it into pieces of a moderate ſize, and by rubbing in the ſalt with a ſtrong hand, as well as by the application of brine or pickle. When too little ſalt is applied, and proviſions are become tainted or corrupt, there is not ſoda enough to arreſt the ſeptic acid, nor a ſufficiency of muriatic acid extricated to impregnate and preſerve the remainder of the meat. From this ſeptic matter, diſengaged with a portion of hydrogen, phoſphorus, &c. proceeds the diſguſting flavour and ſickly nauſeating fœtor of badly-cured proviſions. Thus I account for the production of a large proportion of the peſtilential air evolved from ſtinking *beef*, *hides*, and *fiſh*, which almoſt depopulated New-York this year. An alkali could have ſtaid the whole miſchief.

Soda may be thus conſidered not merely as an antiſeptic, but as a ſubſtance remarkably friendly, and even neceſſary to the conſtitution of the more perfect animals, and eſpecially of man. We are now able to judge a little more clearly and conſiſtently concerning the operation of the various preparations of that alkali upon the alimentary canal. The neutral ſalts, with this baſis, are among the moſt mild and agreeably-efficacious articles of the ſhops. The *tartarit of ſoda* (Rochelle ſalt) is an elegant remedy, and one which I have employed with much ſatisfaction to keep the inteſtines free from noxious matter, in our peſtilential and other fevers. The *phoſphat of ſoda* (ſoda phoſphorata) is, perhaps, a yet more elegant medicine, readily ſoluble in water, eaſy to be taken, and not difficult to be decompounded in the bowels. The *carbonat of ſoda*, diſſolved in water, and taken into the ſtomach at the rate of from four to eight grains in two or three hours, is a moſt gentle and efficacious remedy in dyſenteries, and in cholera infantum. If teneſmus is violent, clyſters of ſoda often afford almoſt inſtant relief. Laudanum, if neceſſary, may be joined with it in both caſes. Indeed, in the three enumerated forms, ſoda is capable of attracting the ſeptic acid, which, no doubt, is a frequent exciting cauſe of dyſentry. The *ſulphat of ſoda* is the WONDERFUL SALT OF GLAUBER, and familiar to phyſicians, and indeed to houſekeepers: I ſhall, therefore, only remark upon it, for the information of chemiſts, that, contrary to the arrangement in the common tables of elective attractions, M. CHAPTAL has ſhewn, (Memoire ſur la Decompoſition a Froid, &c. 1 Memoires de Chimie, p. 47.) that the nitrous (ſeptous) acid can decompound the vitriolic ſalts, that is, thoſe ſaline compoſitions which contain the ſulphuric acid: and he enumerates by name Glauber's ſalt, or ſulphat of ſoda, as one of the vitriolic ſalts capable of being thus decompounded. The *muriat of ſoda* has likewiſe, of late, been highly extolled as a valuable anti-dyſenteric remedy.

But what ſignifies it to enlarge on the preferableneſs of this alkali, in medical preſcriptions, to either of the other alkalies? The very name it now bears is SODA, a word ſynonimous with *pyroſis* and *cardialgia*, and ſignifying that diſorder in the ſtomach called, by the ſpeakers of the Engliſh language, "heart-burn." When I firſt became acquainted with it, the officinal name was *ſal ſodæ*, or *ſalt for the heart-burn*. Latterly, the name "ſoda" has been transferred from the diſeaſe to the ſubſtance uſed in curing it; in which it has been ſo firmly eſtabliſhed ſince its introduction into chemical nomenclature, that many of the younger claſs of of modern inquirers have to find out, by fearching their dictionaries, that foda had ever any other meaning than the one it at prefent bears. It will appear from thefe remarks, that foda was, a confiderable time fince, thought a good thing for the alimentary canal, and the reafon of this was what I have attempted to affign and illuftrate.

This theory of bile and fea-falt will be called vifionary by fome, perhaps the greater part of thofe who perufe it, efpecially if they glance at it fuperficially. To this treatment reformers muft fubmit. Their whole employment is like pulling againft the tide, and fometimes beating againft the wind too. They fhould remember the words of the poet, and be meek:

> "Truths would you teach, or fave a finking land,

> "All fear, none aid you, and few underftand."

---

### *A further Statement of the Cafe of Elizabeth Thompfon, upon whom the Cæfarean Operation was performed in the Manchefter Lying-in Hofpital; in addition to that publifhed by* Mr. Wood, *in the Memoirs of the Medical Society of London, Vol. 5th, by* Charles White, *and* Richard Hall, *Men-midwives Extraordinary;* George Tomlinson, *and* John Thorp, *Men-midwives in Ordinary to that Charity.*

Manchester, *November* 8, 1799.

As a difference of opinion has taken place before the public, between two medical gentlemen, on the fubject of a cafe of Cæfarean operation lately performed in the Lying-in Hofpital at Manchefter, and the facts of it being varioufly reprefented by them, the men-midwives of that inftitution feel it their duty to the profeffion, to ftate their opinion and the circumftances of this interefting cafe.

On Monday the 24th of June laft, about one o'clock P. M. Mr. White received a letter from Mr. Ogden, a furgeon at Afhton-under-Line, which we have fubjoined, together with Mr. White's anfwer.*

About two o'clock, Elizabeth Thompfon was brought to the Lying-in Hofpital

---

* Mr. Ogden's Letter.

CHARLES WHITE, Efq. King-ftreet, Manchefter.

Dear Sir,

Though I am not acquainted with all the rules of the Lying-in-Hofpital, yet, I truft, I am acting in conformity with their fpirit, in fending the poor woman, who is the object of this addrefs, to the Charity in queftion. I have no doubt, you will receive her with cheerfulnefs; and tho' I am afraid there will be much, both of difficulty and danger in the cafe, yet it will be fome confolation to me, to reflect, that every poffible care will be taken of the poor patient; living as fhe does, at a diftance from all obftetrical affiftance, it would be impoffible, under all the circumftances of the cafe, to render her all the neceffary aid and accommodation at home: humanity therefore requires me to act as I am doing. Permit me further

Hospital,* and as soon as she arrived, Mrs. Turner, the matron and midwife, sent for Mr. Wood, the man-midwife in ordinary, for the week, who saw her about three o'clock. He finding the case a very difficult and dangerous one, desired a consultation, when the men-midwives extraordinary, and in ordinary, were immediately sent for; four of whom attended. Mr. Nansan was at Buxton, and Mr. White was gone a few miles out of town; but a messenger was dispatched after him, to request his attendance. He immediately came to town, and arrived at the hospital about half past eight o'clock; where he found Messrs. Hall, Tomlinson, Wood, and Thorp. After having examined the poor woman, their opinions were taken separately, and given to Mr. Wood, without any previous communication with each other; when they were unanimous, that the pelvis was so much distorted, that none of them could perceive either the child, the *os tincæ*, or any part of the uterus; that nothing but the Cæsarean operation could give any chance either to the mother or the child, and that no time ought to be lost in performing it.—The pulse, previous to the operation, beat 120 in a minute.

The plan laid for the operation, was, to pay no regard to the epigastric artery, as it could be of no consequence in a large wound, to

---

further to request, that, should the Cæsarean Section be deemed expedient, in the present case, you will inform me of it, in order that I may be present at the operation.

With becoming respect, I remain,

Dear Sir, your most obedient servant,

*Ashton, 24th June,* 1799. JAMES OGDEN.

Mr. White's Answer.

Mr. OGDEN, Surgeon, Ashton-under-Line.

Dear Sir,

Immediately upon the receipt of your letter, yesterday, I sent it down to the Lying-in-Hospital, and along with it, a recommendation for the poor woman; but she was not then arrived, and I was obliged to go out of town; but Mr. Wood, under whose care she fell, finding the case a very deplorable one, called a consultation, and a special messenger was dispatched after me, to request my attendance. When I arrived, betwixt eight and nine o'clock, I found Messrs. Hall, Tomlinson, Wood and Thorp; we were all unanimous, that no relief could be obtained for the poor woman, except by the Cæsarean Operation, which she consented to without the least hesitation, and it was performed by Mr. Wood without any accident or disappointment. The child is alive and hearty, and was christened this morning by the name of Julius Cæsar. The poor woman has had a very good night, and is as well in every respect as can possibly be expected. She bore the operation without a complaint, and says it was a much easier labour than her former one.

It gave us all great concern that we had not time to send for you, but the lateness of the hour when the consultation took place, and the great consequence it might be of to the poor woman to have delayed the operation, we hope will be a sufficient apology for not requesting your attendance, but we shall be extremely happy if you will have the goodness to attend our consultation at the Hospital to-morrow morning at a quarter before seven o'clock.

*Manchester,* I am, yours most sincerely,

*June 25th,* 1799. C. WHITE.

Mr. Ogden attended the consultation at the time appointed.

* We find, upon inquiry, that she was brought in a cart, placed on a feather bed which was slung with cords, in imitation of a hammock; but the mode which we recommend for conveying women in labour, from a distance, is a sling, carried by two men; it is easily constructed in any country place, with two poles and a couple of sacks.

One upon this construction is kept in the Hospital, for that purpose, and may be had when applied for.

men accuſtomed to perform operations, and who knew the uſe of the needle and tenaculum. The place of election for the inciſion appeared to them to be, where the child could be moſt eaſily perceived, where they were not likely to meet either with inteſtines or placenta, or any intervening ſubſtance of conſequence. The operation was well performed, and with great ſteadineſs, by Mr. Wood, in the preſence of Meſſrs. White, Hall, Tomlinſon, and Thorp, of Mr. Chippindall, the apothecary to the charity, Mr. Barlow, Mr. Wood's aſſiſtant, and Mrs. Turner, the midwife. The epigaſtric artery was not wounded; the inciſion in the uterus was not more than ſufficient to extract the child; there was no hæmorrhage to threaten life, or to impede her recovery; and what blood was ſhed into the cavity, was taken up by a ſponge. The child lay upon its right ſide, with its head, in the neck of the uterus, reſting on the fourth vertebra of the loins, and on the right ilium and pubis, completely above the ſuperior aperture. Whoever will be at the trouble of applying a fœtal ſkull to this diſtorted pelvis, will be convinced, that it could not take any other poſition, the head could not deſcend ſo low *as to be jammed in between the bones of the pelvis*; it could not even deſcend ſo low as the fifth vertebra of the loins; ſo that the *cervix uteri* appears to have been forced, at every pain, againſt the *os innominatum* on the right ſide, and the fourth vertebra of the loins. *The natural ſhape of the head was not at all changed* from its round form to an oblong or ſugar-loaf form; and it is impoſſible that it ſhould have ſo changed, becauſe the ſuperior aperture was too narrow, and too diſtorted, to admit of its deſcending through any part of that aperture; and as the bones of the pelvis could not give way, the child's head, by every labour pain, would drive the *cervix uteri* againſt the ſolid bone, and produce an alarming degree of contuſion—thence the danger.

We attended the patient three times a day, as long as ſhe lived; and we are ſatisfied, that the after-treatment was right and proper in every reſpect. But thus far we may ſay, that, though ſhe did not loſe ſo much blood as to endanger her life or impede her recovery, we were of opinion, that, taking more, either generally or locally, would, on account of her previous indiſpoſition, have occaſioned too great a debility. We thought ſeventeen hours after the operation early enough to inject a clyſter; as the inteſtines had been ſufficiently emptied, a little while before the operation, by a violent diarrhœa. *We never, at any one time, thought her free from danger.* She died an eaſy death, and was extremely thankful for what had been done for her; and we heſitate not to give it as our opinion, that, performing the operation was the greateſt act of humanity that could be done to the poor creature, who was labouring under as excruciating pains as we ever knew to fall to the lot of a human being.

The body was opened by Mr. Wood, in the preſence of the Medical Committee of the hoſpital. The gentlemen who attended were, Dr. Cowling, Meſſrs. White, Hall, Thorp, Foxley, Brigham, Ollier, and Clough; Mr. Chippendall and Mr. Barlow were alſo preſent.

There were about ten ounces of bloody ſerum, with a little coagulated blood, (not more than an ounce) in the cavity of the abdomen.

The inteſtines were much diſtended with wind; but very few fæces were contained in them, and none of them were hardened.

There was no appearance of peritoneal, or inteſtinal inflammation; no no inflammatory exudation; no flakes of coagulable lymph; nor any extravafated fluid, of the nature of milk, refembling unclarified whey, containing flakes of curd-like matter, adhering to the inteftines; nor had the inteftines formed any adhefions; nor were there any unfavourable appearances about the wound in the uterus, or in that of the integuments.

The uterus was taken out of the body, and the *os tincæ* was found dilatedto about two inches and a half in diameter; but ftill nothing could be difcovered that could poffibly account for her death, until it was cut open, when the infide being carefully wafhed with a fponge and warm water, a gangrene appeared quite round the infide of the neck of the uterus, rifing higher in nearly a circular form, in the fore part, where the child's head was believed to have preffed it againft the elevated part of the *ossa pubis*. This mortification* in the neck of the womb, which was totally unconnected with the incifion in that organ, we confider as fufficient in itfelf to account for the death of the woman.

As soon as the uterus and pelvis were removed from the body, Mr. Wood, fent round to the phyficians and furgeons of the Manchefter Infirmary, and to feveral other medical gentlemen, who had not attended the diffection, to requeft their attendance at the hofpital, where the uterus and pelvis were left for their infpection. In the courfe of that day, the following gentlemen attended, and faw the uterus and pelvis.

Dr. Holme, phyfician to the Infirmary; Dr. Hull; Mr. Bill, Mr. Killer, Mr. Ward, and Mr. Hamilton, furgeons to the Infirmary; Mr. Taunton, furgeon to the Cornwall Fencibles, at the Barracks; and Mr. William Henry.

---

* Mortification frequently takes place without any inflammation preceding; and, that mortification of the uterus will happen, without much previous warning, will appear from a cafe which Mr. White publifhed in the appendix to his Treatife on the Management of Pregnant and Lying-in Women, ed 5th. page 448, of a lady who died on the 8th day after delivery, of a mortification of the uterus, but had made no complaint till within fix hours of her death.

The uterus is an organ which is not abfolutely neceffary to life, fince many animals have been known to live after it has been taken out. Ætius and Paulus Ægineta fay, that they have known even women recover, when the uterus had been extirpated on account of an inverfion; and the fame is mentioned by Paré. A very interefting cafe of this kind is related by Prof Wrifberg of Göttingen (Com Soc. Reg Sc. Gott tom. 8.) Mary Dorothy Ude was delivered by a midwife of her firft child on the 5th of June, 1780, who ufed fo much violence in attempting to bring away the placenta, that fhe inverted the uterus, and immediately afterwards cut it away with a knife, exactly in the part where it is connected with the vagina. The poor woman was greatly endangered by the hemorrhage, but recovered completely. In September 1786, the aperture, which before that time would admit a finger, was become almoft clofed.

Dr. Holme, Phyfician to the Manchefter Infirmary, faw and examined this woman in the year 1790, at Göttingen.

N. B. Cafts of the pelvis, taken in plaifter of Paris, by Mr. Sardini, may be had of him in London, and of Mr. Chippindall, the Apothecary at the Lying in Hofpital in Manchefter, where practitioners may have an opportunity of convincing themfelves of the utter impoffibility of delivering, where there are fuch diftorted pelves, by any other means than the Cæfarean Operation. We have the fatisfaction to fay, that the child is very ftrong and hearty.

---

From the prior account given by Mr. Wood, in the Memoirs of the London Medical Society, Vol. V. p. 475, relative to the dimenfions of the pelvis, in the fubject here alluded to, it appears, that the largeft circle which could be formed in any part of the fuperior aperture, did not exceed *one inch* in diameter.—Ed.

Dr.

Dr. Percival called on Mr. White, in order to have gone with him to the hospital; but not finding him at home, and other circumstances occurring, prevented the Doctor from seeing the uterus at that time, but he afterwards saw it at Mr. Wood's.

Mr. Simmons, in a note to Mr. Wood, thanks him for his polite attention, but declines in the present instance accepting his invitation.

The Cæsarean operation has been succesfsully performed in Ireland; in the West Indies; frequently upon the Continent of Europe; and within these six years at Blackrod in this county. The only matter of dispute, in this last instance, is, whether the uterus was cut open with a knife, or was burst. The operator says it was cut open, the assistant says it was burst. We are not aware that a lacerated wound has any advantage over an incised one, except in preventing hæmorrhage, which we do not find to be a material objection to the operation.

We believe that the Cæsarean operation, and cutting the symphysis of the pubis, have frequently been unnecessarily performed upon the Continent, in cases where an English Accoucheur would never have thought of having recourse to either; and, on the contrary, we believe that many lives have been lost in this kingdom, for want of the Cæsarean operation being performed, and that some have been lost from its having been too long delayed. But, we should be much concerned to find it ever become other than a rare operation.

It should never be resorted to, when milder means will answer; nor should the life of the child be put in competition with that of the mother; nor should it in any case be performed, without a consultation of the most eminent practitioners in the neighbourhood.

Many women's lives have been saved by the crotchet in distorted pelves; but there are some cases, where it cannot possibly be used; six having occurred in this town, within our knowledge, in which the delivery could not be accomplished by means of this instrument, and the Cæsarean operation, not being had recourse to, all the mothers and children perished.

---

### *To the Editors of the Medical and Physical Journal.*

GENTLEMEN,

IN the fifth volume of the London Medical Memoirs, just published, there is a paper from Mr. Wood, of Manchester, surgeon, relating a case of the Cæsarean operation, which he has lately performed in the Lying-in hospital of that town, and in which it was my fortune to be somewhat concerned. As Mr. W. in this paper, has drawn some inferences which may, and which I suspect were *meant* to, convey a censure as well upon Mr. Simmons as myself, I trust you will give a place, in the next number of your Journal, to the following statement and remarks.

On Monday the 24th of June last, between two and three o'colck in the morning, I was called up to go and visit the wife of John Thompson, of Hazlehurst, in this parish, as being then in labour. The distance from my house is about two miles. I went immediately; and on my arrival there, was informed that the poor woman, who was quite quite a ftranger to me, had then been in labour about two hours. After examining her per vaginam in the firft inftance, and interrogating her afterwards refpecting her former labours, her general ftate of health, &c. I informed her hufband that fhe could not be delivered either very foon or very eafily, and that I wifhed to have a confultation on her cafe with a friend of mine from Manchefter. I defired him to get a horfe as foon as poffible, and follow me home, where I would have a letter re.dy for him accordingly. He did fo. This letter I addreffed to Mr. Simmons, who came, and faw the woman along with me before nine o'clock, A. M. After a cautious examination, Mr. S. was of opinion that the cafe was one of Dr. Ofborne's crotchet cafes. The woman's pains at this time recurred very regularly; her fkin was cool, her pulfe calm, and, abftracted from the obvious deformity of the pelvis, there was nothing to excite either alarm or fufpicion. As it was impoffible for Mr. S. to undertake the cafe at fuch a diftance from home, and as I had many reafons for declining it myfelf, the Lying-in hofpital occurred to me as the moft proper for the occafion. I immediately communicated this fuggeftion to both the hufband and his wife, who acceded to it without hefitation. I then ordered a cart to be fitted up with ropes, in fuch a manner that a bed could be fufpended by them without refting on the cart; and on a bed flung in this way the woman was conveyed to the Lying-in hofpital, and, as the hufband informed me foon afterwards, with more eafe, convenience and expedition than could have been expected. She arrived at the hofpital about one o'clock, P. M. Along with the patient I fent a letter to Mr. White, as fenior accoucheur to the charity, requefting admiffion for her: and fearing Mr. W. might not be at home on its arrival, I fent my letter open, with a verbal inftruction to the bearer, that, in fuch cafe, he fhould take it to Mr. Hall (the next in feniority to Mr. W.), who would be able to fee its contents and act accordingly. I requefted farther, in my letter to Mr. W. that fhould the Cæfarean fection be deemed expedient, he would let me know, in order that I might be prefent at the operation. I heard no more of the cafe until ten o'clock in the evening of the next day, the 25th, when I received a letter from Mr. W. dated the fame day, ftating, that the Cæfarean operation had been performed the evening before, at nine o'clock; that the child was alive and healthy, and that the mother was in a very promifing fituation. And as an apology, I fuppofe, for not advifing me of the operation prior to its being done, Mr. W. expreffed a wifh to fee me at the hofpital the following morning at feven o'clock, being the hour agreed upon for confultation. Believing the poor woman would certainly die, in confequence of the operation, and being extremely anxious to fee her while living, I fet out for Manchefter the fame night, and arrived at the hofpital foon after midnight. I met the gentlemen of the hofpital the next morning, the 26th, at feven o'clock, as defired, when their opinion ftill was, that if a paffage could be procured per anum, the patient would do well. It was mentioned too, incidentally, that at fome period fubfequent to the operation, (I do not remember precifely when, and therefore I dare not fay) her pulfe had been below 100. I mention thefe circumftances merely to fhew, that, in the opinion of the accoucheurs of the hofpital, no inflammation of *any fort* exifted either *before* the operation *or on the day following it*, and that *even on the morning of the fecond day after the operation*, they were fcarcely, if if at all, alarmed for the patient's ſafety. My own opinion, it is true, was widely different from theirs, and I declared it to them accordingly. I remained in town a few hours longer, purpoſely to watch the progreſſion of ſymptoms; and when I ſaw her for the third and laſt time, about one o'clock, I did not conſider her recovery as a poſſible occurrence. She died about 36 hours afterwards.

In giving an account of the diſſection, Mr. Wood attributes the woman's death to a gangrene about the cervix uteri; and this gangrene, he chooſes to ſay, was occaſioned by the preſſure of the child's head, as impelled merely by the pains of labour, aſſiſted, perhaps, by ſome *rugged* concuſſions of her bed, while travelling in the cart. Now when it is conſidered, how often caſes of this ſort have been *undecided for many days ſucceſſively*, without the patient's ſuffering any material injury ſimply from the delay, during all which time it has been uſual, alſo, to employ ſome other violent means to accompliſh the delivery, I put it to the candour and good ſenſe of the medical world at large, whether it is at all probable, that the woman's death, in queſtion, was occaſioned by the cauſes as accounted for by Mr. Wood. But I will *ſuppoſe* the caſe to be as Mr. W. wiſhes us to believe, that a mortal inflammation of the uterus was excited, no matter how, *before* the operation, pray what can excuſe his performing ſo ſerious an operation under ſuch a circumſtance? Had a diſeaſe ſo truly formidable *no* ſymptoms, either general or characteriſtic? Is it even poſſible for ſuch a diſeaſe to be excited in ſo few hours, in the way ſuggeſted, and to elude diſcovery? If ſo, I ſhould be glad to know from Mr. W. or from his aſſociates, *when* we can be certain that ſuch a diſeaſe does *not* exiſt? and whether, on ſuch a ſuppoſition, we can ever be juſtified in performing the operation at all, *except as a preventative before the coming on of labour?* Mr. Wood chooſes to ſay farther, that if the operation had been performed ſooner, and at the patient's home, ſhe would probably have recovered. Suppoſing this to be true, it is very much to be lamented that the poor woman ſhould have been *eight* hours in the hoſpital, before the operation was performed. The public, I am confident, will eaſily ſee that no time was loſt in getting her there. I do not wiſh to ſay any thing reſpecting the *treatment* of the caſe. Mr. Wood's account of it is before the public: it is all the account which the public is likely to receive, and, for aught I know, it may be correct as far as it goes. But I muſt be permitted to aſk, if it was of ſo much importance to empty the bowels very ſoon after the operation in queſtion, as undoubtedly it was, why was nothing of this ſort done *before* the operation? The operation was performed on the Monday evening at nine o'clock, and the patient had had no ſtool from the afternoon of the day preceding. I do not mean to infer much from this omiſſion, though I do think it ought to have been moſt ſtudiouſly guarded againſt. It is very unphiloſophical, to ſay the leaſt of it, to ſeek for, or to reſt upon, minor circumſtances as occaſioning the woman's death, when we have *one* ſo abundantly ſufficient to account for it, and which has occaſioned the death of every other woman in this country in the ſame ſituation;—*I mean, the operation itſelf.*

Having thus ſhewn that the poor woman's death not only was not, but could not be, occaſioned in the way, and as explained by Mr. Wood, the only inference which I can draw from the caſe is, and the public at large, I am perſuaded, will draw the ſame—*that the Cæſarean operation*

*ought*

*ought never to be performed at all, in any caſe whatſoever, during the life of the mother.*

I am, Gentlemen,

Your moſt obedient ſervant,

JAMES OGDEN.

*Aſhton-under-Line,*

*Nov.* 5, 1799.

P. S. Since writing the above, I have ſeen the ſtatement, as drawn up by Mr. Simmons, and which he intends, I believe, for publication in your Journal. It is only neceſſary to obſerve, reſpecting Mr. S's account, that the facts ſtated in it are ſtrictly true.

### *Miſcellaneous Extracts.*

Citizens Dumeril, Chaussier, and Dumas have communicated to the *Philomatic Society*, various plans of an Anatomical Nomenclature. The firſt plan is founded on the termination of nouns; and the author adopts, as the radical, that name of the bone which is ſurrounded by the parts to be deſcribed; and the termination alone indicates, whether the name belongs to a muſcle, an artery, a vein, or a nerve, &c. The methodical claſſification and nomenclature preſented by the ſecond Citizen poſſeſs great advantages over thoſe hitherto used; as more uniformity in the deſignations has long been a deſideratum. The Nomenclature propoſed by Cit. Dumas, differs but little from that of Citizen Chauſſier; it is compoſed of names which the author has endeavoured to define, but they are often formed of words rather difficult to be remembered. The eight chapters in which he compriſes the introduction to this work, are written with clearneſs and preciſion, and abound in philoſophic reflections.—*Rapport Général des Travaux de la Societé Philomatique de Paris,* 1798. vol. i. p. 143.

Citizen L'Eveille has laid before the Society, ſeveral reports relative to the inquiries made by him in conjunction with Citizens Larrey and Dumeril, to authenticate the obſervation made by Dr. Soemmering, profeſſor of anatomy at Mentz, on the retina of the human eye. He has diſcovered a yellow ſpeck ſituated in the axis of the eye, near the inſertion of the optic nerve, and which has an aperture in its centre, not before obſerved by anatomiſts. L'Eveillé has found this ſpeck, with its aperture, in ſeveral human ſubjects; he imagines it to be deſtined for the modification of light, when its impreſſion on the retina is too vivid; a circumſtance of which Soemmering takes no notice.—*Ibid.* p. 144.

The ſame member has communicated to the Society, an obſervation which he made reſpecting an infant born with an imperforate anus; and whoſe rectum communicated with the bladder. The ſurgeons had in vain attempted to make an artificial anus in this ſubject.—*Ibid.* p. 145.

He alſo gave an account of a fœtus, ſix months old, having only one eye, ſituated immediately above the noſe: This eye contained two tranſparent corneâ, ſeparated from each other by two opaque corneâ; it had had two optic nerves; but he could not difcover the fmalleft trace of the nafal cavities.—*Ibid.*

The fame anatomift relates the diffection of a fubject twelve years of age, in which he found the pericardium adhering to the heart, and entirely in a ftate of fuppuration. This fubject had died in confequence of frequent convulfions over the whole body; and had fuffered much pain on being touched.—*Ibid.*

Citizen Halle has given a defcription of an extraordinary fœtus, in which the brain had formed a hernia acrofs the cranium. On the right fide, the ribs were feparated from the fternum; the whole of the right arm was formed of two bones end to end, terminating in a fingle finger. The clavicle was found articulated with the os humeri and the fternum. This fœtus had no fkin on its belly. All the inferior vifcera were difplaced from its abdomen; nor had it a gall bladder: it was furnifhed with only one leg.—*Ibid.* p. 147.

Citizen Cuvier informed the Society, that he had received a defcription of a fubject which had neither head nor heart. Its body was divided into three lobes, two of which reprefented the thighs and legs, and the third the trunk. Citizen Brongniart had formerly given the Society an account of a child born without head, arms, heart, lungs, or ftomach, and wanting feveral other parts effentially neceffary to a fœtus. —*Ibid.*

Citizen L'Eveille opened a female fubject that had been treated for an internal complaint, at the Hotel Dieu, at Paris. She was eight months gone with child, and the ovarium of the right fide formed a confiderable tumour, containing a mole as big as a hen's egg, and a well-formed fœtus, apparently three months and a half old; its various limbs, as well as its placenta and umbilical cord, could be eafily diftinguifhed.— *Ibid.* p. 146.

Citizen Brongniart read a memoir to the Society, on his diffections of feveral apes; *Simia cinocephalos, capufina, æthiops, memon, & fabea.* He compared the myology of thefe fpecies together, and with that of man. Hence he concludes, that apes have a greater number of mufcles than the human fpecies; that thefe mufcles are more flefhy, and often more elongated; but that, in general, the tendons and aponeurofes are much lefs; that the mufcles of the pelvis, and of the pofterior extremities, are fo fituated as to oppofe themfelves, in apes, to the long-continued vertical pofition; but that their form and arrangement enable thefe animals to jump and climb with extraordinary agility. In fhort, he is of opinion, that apes refemble quadrupeds in their mufcular organization much more than they refemble man.—*Ibid.* p. 150.

Citizen Cuvier read a memoir on the hearing faculty of whales, and prefented to the Society the auricular bone of one of thefe animals. This bone does not form an effential part of the cranium, but is fufpended by mufcles and ligaments. He diftinctly faw, in the fœtus of the whale, the circular canals, the exiftence of which in cetaceous animals, has been denied denied by Camper. To this memoir is added a table of the characters of the internal ear, in all the classes which are provided with that organ; whence it appears, that the essential part of the ear is a kind of transparent gluten, into which the auditory nerve seems to resolve itself.—*Ibid.* p. 148.

---

Citizen LATREILLE transmitted to the Society, some observations on the organs of generation of the *Julus complanatus*, L. The male of this insect has sixty feet, and the female sixty-two. Instead of the two which are wanting in the male, are two yellow, transparent, and projecting feet, which belong to the organs of generation, and are not externally perceptible. In the female, the genitals dilate only in coition, being imperceptible at any other time: these insects copulate front to front.—*Ibid.*

---

Citizen L'EVEILLE made a report on a new attempt to perform the Cæsarean section. The woman who underwent this operation died immediately. On dissecting the body, Citizen L'Eveille found the aperture of the uterus exactly parallel to that of the abdominal integuments, contrary to the opinion of several authors, who assert, that on the birth of the child, the uterus returns to its natural situation.—*Ibid.* p. 152.

---

The same member mentioned an observation he had made on an *aneurism* of the aorta. On the left side of the breast, a tumour appeared about five inches long, and three and a half broad: it had very frequent pulsations, resembling a settled palpitation; the pulse on the side of the aneurism was almost imperceptible; the patient soon expired, being suffocated by the quantity of blood discharged from the tumour, which instantly disappeared; the intercostal muscles had degenerated into a cartilaginous substance; the third true rib was entirely destroyed by a caries which had attacked the second and fourth ribs. The author observes, that it is very difficult to determine the cause of this disease, which of late has become very frequent.—*Ibid.* p. 152.

---

Citizen LACROIX read an observation on an extra-uterine conception. The patient, only by the suppression of her menses, discovered her pregnancy, five months after which she perceived some internal motions. At the end of seven months, the fœtus ceased to move. A considerable discharge, attended with painful symptoms, induced Citizens Baudeloque and Lacroix to suspect an extra-uterine conception. This woman died soon afterwards, and, on opening the body, they found the large ligament and the Fallopian tube blended in a sac, formed by the epiploon; there was likewise a fœtus lying towards the left side of the belly, in the usual posture; the uterus was in its natural state; the dilatation of the Fallopian tube, in which the fœtus had grown, was one inch from it; the skin and the cellular membrane of the child had changed their nature, and when pressed by the thumb, appeared like fat; they were similar to the fat of dead bodies found in the cemetery of the Innocents, except that, in the analysis, they did not afford any ammonia.—*Ibid.* p. 156.

---

Citizen L'EVEILLE communicated some observations on a tetanus which succeeded a wound of the finger. To avoid a fall from a ladder, the patient caught a hook, by which he remained ſuſpended: this made a deep wound in the anterior part of the middle finger, which cicatrized in a few days; but ſoon afterwards the patient felt ſuch acute pains in the face, and diaphragm, that he was obliged to apply to the hoſpital. The tetanus now appeared; his jaws were completely locked; the recti muſcles were rigid and projecting; his back was concave, and his breaſt convex. On touching his belly, the muſcles of the neck became contracted. Citizen PELLETON cured this man in twenty-one days, by the frequent uſe of baths, and the adminiſtration of laudanum.—*Ibid.* p. 153.

---

Citizen DUMERIL communicated the reſult of an experiment he had recently made. It had been formerly obſerved, that when the bones became accidentally diſlocated, new articular cavities were formed in the place where they touched. An attempt was made to repeat this experiment upon animals. After having diſlocated the thigh of a young dog, and amputated the extremity of the femur, below the trochanter, the animal was allowed to walk about. A ſhort time after, on opening the body, it was obſerved that a new articular cavity was formed; from this fact it was inferred, that in a caſe where a caries would render it neceſſary to extract a part of human bone, the carious part might be removed, as the re-union of the two healthy parts would form a new articular cavity, and the patient would experience no other inconvenience than the ſhortening of the limb.—*Ibid.* p. 154.

---

Citizen LARREY read an obſervation on a conſiderable tumour, the nature of which was miſtaken before and after the death of the patient. The origin of this diſeaſe was aſcribed to flying rheumatic pains which ſettled in the knee. The patient having had a fall on that part, felt a crack in the joint, and was unable to riſe; inflammation ſucceeded, and, notwithſtanding the aid of medicine, he daily grew worſe. Citizen PELLETAN, who ſuppoſed this diſorder to be a *ſpina ventoſa*, propoſed the amputation of the thigh: ſome other practitioners conſidering the tumour as lymphatic, determined to open it with a trochar. Two inciſions were made, from which iſſued a fetid gas and ſanious ichor, but the patient died the ſecond day after the operation. On opening the body, the femur, near the condyles, was found in blackiſh fragments, and corroded by the caries; the marrow was black and diſorganiſed; the perioſteum partly detached, and the ſurrounding ſoft parts reduced to a ſpongy ſubſtance, which had been penetrated by ſeveral ſplinters of the thigh bone.—*Ibid.* p. 155.

---

### *Domeſtic Intelligence.*

A few botanical friends have formed a plan of a popular work, by which they propoſe to reſcue the *Science of Botany* from the libraries of the learned, and to ſubſtitute for all the *technical* terms, hitherto uſed in elementary works, appoſite Engliſh names and phraſes, ſuch as are eaſily underſtood by every reader of a cultivated mind. Their principal aim is, is, to *inveftigate, afcertain, and apply the properties of all native plants to fome ufeful end, or purpofe.* While other botanifts have fuccefsfully laboured to furnifh us with accurate defcriptions and faithful delineations of plants, the aim of this Society will be directed to the *more practical*, or *economical part of Botany:* by pointing out, in familiar language, whether, and in what degree, our indigenous plants may afford *fuel, meal, oil, falt, foap, colouring matter, glue and pafte, rofin, wax, paper, chocolate, and tobacco,* or whether they may be more profitably employed in the different proceffes of fermentation, for making *wine* or *vinegar,* brewing *beer,* diftilling *fpirits;* or for manufacturing *linen, cotton,* and *filk cloths;* for the various arts of *dying, varnifhing, tanning, &c.* It is fuperfluous to mention that this comprehenfive work, fo much wanted in an age of the ftricteft economy, will appear under the aufpices of the moft illuftrious characters in this country; and, as a farther account of it is intended to be given in future Numbers of our Journal, we fhall only add, that it is to be entitled, THE BRITISH ENCYCLOPEDIA BOTANICA.

---

Mr. PARKINSON, whofe Medical Admonitions were mentioned in our Journal for May and July, has, we underftand, in the prefs, befides the *Memoranda Chemica,* which we announced fome time fince, a popular medical work, intended for that clafs of fociety who can feldom purchafe medical aid. The health and happinefs of thefe he means to promote by pointing out, in a familiar lecture, rules for the profecution of labour and of recreation; the injuries to health arifing from irregularities; the duties due from parents to children; the means of removing the firft fymptoms of difeafe, &c.

---

Dr. DARWIN's new work, entitled, "Phytologia, or the Philofophy of Agriculture and Gardening, with the Theory of Draining Moraffes, and an improved Conftruction of the Drill Plough, is fo far advanced at the prefs, that it may be expected before Chriftmas; it will form a large quarto volume, illuftrated with plates.

---

Dr. BEDDOES, who is indefatigable in the caufe of ufeful fcience, announces the early publication of a popular medical work, in which he intends to unfold that portion of the order of Nature, which regulates the movements of the animal machine, and along with the principles, to ftate explicitly thofe practices relative to the prefervation and recovery of health, upon which alone unprofeffional readers can fafely venture. Dr. Beddoes benevolently "wifhes to render health a main object of education; to deter the ignorant from tampering with the fick; and to curtail the dominion of empirical impofture." The firft number of this work will appear after Chriftmas, and the whole will be of confiderable extent, and be enriched with engravings.

CRITICAL

